# Editorial: Novel molecular targets and therapies for pediatric extracranial solid tumors

**DOI:** 10.3389/fonc.2025.1695440

**Published:** 2025-09-19

**Authors:** Paola Angelini, Aditi Vedi, Kavitha Srivatsa

**Affiliations:** ^1^ Division of Paediatrics, The Royal Marsden Hospital and Division of Molecular Pathology, The Institute of Cancer Research, Sutton, United Kingdom; ^2^ Department of Paediatric Haematology/Oncology, Cambridge University Hospitals NHS Foundation Trust, Cambridge, United Kingdom; ^3^ Department of Paediatrics, University of Cambridge, Cambridge, United Kingdom; ^4^ Great Ormond Street Hospital for Children NHS Foundation Trust, London, United Kingdom

**Keywords:** paediatric cancer, extracranial solid tumour, innovative therapeutic agents, children, clinical trials

We all dread the moment when we have to tell a family dealing with childhood cancer that there are no more treatment options, and there is no clinical trial available. The *Novel Molecular targets and therapies for paediatric extracranial solid tumours* topic papers well summarize the avenues paediatric oncologists are pursuing to avoid ever having that conversation again, and the challenges they are facing.

Over recent decades, a vast amount of genomic data on paediatric cancers has been generated and made publicly available, with the aim of not only understanding the pathogenesis of disease and relapse, but also identifying therapeutic targets. Ward et al. use a complex methodology, starting from the analysis of a large public genomic database to formulate a hypothesis, which they then validate with traditional methodologies: an elegant example of how this data can be mined for new aims.

Genomic data has so far dominated personalized medicine efforts: national programs in the UK (1), North America, Australasia (2), France (3), Germany, The Netherlands have been developed to perform WGS/WES +/- RNAseq on recurrent tumours, to identify targetable aberrations, in order to offer new hope to patients with very poor prognosis. The results have not always met expectations: while benefit can be proven in a proportion of patients, many still do not have directly targetable aberrations, and even in patients who do have genomic targets, not all are able to access targeted therapies. More recent studies have used expression profile (RNAseq) or *ex vivo* drug screening to identify personalized therapeutic options and predict response to treatment (4), but even this labour-intense, expensive approach has shown limitations, as we lack reliable assays to identify drivers and predict response to treatment in patients. Wragg et al. tackle this problem by developing an innovative zebrafish model. They propose co-clinical studies to compare existing models of rhabdomyosarcoma (zebrafish, *in vitro* and mouse) with respect to their capacity to mimic the patient’s response. Their model has a dual readout, lending it to assessment of both conventional chemotherapy and new agents, many of which have an effect on the microenvironment and vasculature.

Hopefully, future, robust, structured approaches will allow us to predict response in patients, both for the purpose of personalized treatment approaches, and to help select the best candidates for early phase clinical trials. In our topic, Fenwick et al. report the results of the PARC study, a phase I-II study testing the safety and efficacy of arginase depletion in solid tumours. The results are encouraging and the drug will be considered for phase II trials in particular in neuroblastoma, likely in combination with chemotherapy and/or immunotherapy. While this is a successful example of evaluating novel agents in early phase trials, the challenges of these clinical trials in rare diseases such as childhood cancer cannot be underestimated. Thorough pre-clinical studies are required to select the best targets and drugs, based on the disease biology and mechanism of action, and more innovative trial designs, incorporating multiple new agents and exploring new ways to identify individualized therapeutic targets. Platform trials with predefined ‘go’/’no go’ criteria for expansion are a promising step towards this goal of minimising patients being exposed to harmful or ineffective doses of novel treatments. A multi-stakeholder approach with early involvement of pharmaceutical companies and regulatory agencies, as well as patients’ advocates, is also crucial to success of these trials and advancing treatments for childhood cancer

A personalized medicine approach can identify therapeutic opportunities for patients no longer well served by standard treatment protocols, including those with multiply relapsed disease or extremely rare conditions: not by chance, those were not represented in our topic. Rare tumours, renal, and liver tumours are underrepresented in clinical trials, as well as in preclinical research projects, due to the paucity of cases and biological specimens. A global approach can help tackle this problem, as exemplified by the PHITT trial, which facilitated the collection of biological specimens from hundreds of patients with liver cancer, or the Glo-BNHL trial, a major cross Atlantic effort for extremely rare disease patients. Larrosa et al. demonstrate the repurposing of targeted agents beyond their approved clinical indication, providing children with rare disease some hope, based on strong biological rationale. Similarly, He et al. expand our knowledge of accepted age limits for rare cancers, permitting drug repurposing in the context of diseases that are not amenable to being evaluated in large scale clinical trials.

Interestingly, the Chun Yin Chan et al. case report extends the use of targeted agents to a benign condition, ganglioneuroma. While this application remains investigational, and limited to extraordinary cases, as the experience with novel agents accumulates, consideration could be given in situations in which a benign condition is life threatening or life changing because of the anatomical location. Another example here is ALK+ Inflammatory Myofibroblastic Tumour and use of ALK inhibitors to avoid or reduce the magnitude of debilitating surgery.

In this special topic, the breadth of novel therapies is well exemplified, for neuroblastoma, in the review from Alkhazal et al. As more drugs become available, new questions arise: how to prioritize agents, design combinations, individualize treatment, which assay predicts response (and hopefully, survival) the best? Artificial intelligence-based models can possibly help navigate these questions. However, this is best kept for another topic where it can be explored in more depth.

## References

[1] Hodder A Leiter SM Kennedy J Addy D Ahmed M Ajithkumar T. Benefits for children with suspected cancer from routine whole-genome sequencing. Nat Med. (2024) 30:1905–12. doi: 10.1038/s41591-024-03056-w, PMID: 38956197 PMC11271414

[2] Wong M Mayoh C Lau LMS Khuong-Quang D Pinese M Kumar A. Whole genome, transcriptome and methylome profiling enhances actionable target discovery in high-risk pediatric cancer. Nat Med. (2020) 26:1742–53. doi: 10.1038/s41591-020-1072-4, PMID: 33020650

[3] Berlanga P Pierron G Lacroix L Chicard M Beaumais TA Marchais A. The european MAPPYACTS trial: precision medicine program in pediatric and adolescent patients with recurrent Malignancies. Cancer Discov. (2022) 12:1266–81. doi: 10.1158/2159-8290.CD-21-1136, PMID: 35292802 PMC9394403

[4] Lau LMS Mayoh C Xie J Barahona P MacKenzie KL Wong M. *In vitro* and in vivo drug screens of tumor cells identify novel therapies for high-risk child cancer EMBO Molecular Medicine. Eur J Cancer. (2022) 14:e14608. doi: 10.15252/emmm.202114608, PMID: 34927798 PMC8988207

